# Progenitor Cells Play a Role in Reinstatement of Ethanol Seeking in Adult Male and Female Ethanol Dependent Rats

**DOI:** 10.3390/ijms241512233

**Published:** 2023-07-31

**Authors:** Hannah A. Nonoguchi, Michael Jin, Rajitha Narreddy, Timothy Wee Shang Kouo, Mahasweta Nayak, Wulfran Trenet, Chitra D. Mandyam

**Affiliations:** 1 VA San Diego Healthcare System, San Diego, CA 92161, USA; 2 Department of Anesthesiology, University of California San Diego, San Diego, CA 92161, USA

**Keywords:** ethanol self-administration, CIE, medial prefrontal cortex, hippocampus, tight junction proteins, microglia

## Abstract

Female and male glial fibrillary acidic protein-thymidine kinase (GFAP-TK) transgenic rats were made ethanol dependent via a six-week chronic intermittent ethanol vapor (CIE) and ethanol drinking (ED) procedure. During the last week of CIE, a subset of male and female TK rats was fed valcyte to ablate dividing progenitor cells and continued the diet until the end of this study. Following week six, all CIE rats experienced two weeks of forced abstinence from CIE-ED, after which they experienced relapse to drinking, extinction, and reinstatement of ethanol seeking sessions. CIE increased ED in female and male rats, with females having higher ethanol consumption during CIE and relapse sessions compared with males. In both sexes, valcyte reduced the levels of Ki-67-labeled progenitor cells in the subgranular zone of the dentate gyrus and did not alter the levels in the medial prefrontal cortex (mPFC). Valcyte increased ED during relapse, increased lever responses during extinction and, interestingly, enhanced latency to extinguish ethanol-seeking behaviors in males. Valcyte reduced the reinstatement of ethanol-seeking behaviors triggered by ethanol cues in females and males. Reduced seeking by valcyte was associated with the normalization of cytokines and chemokines in plasma isolated from trunk blood, indicating a role for progenitor cells in peripheral inflammatory responses. Reduced seeking by valcyte was associated with increases in tight junction protein claudin-5 and oligodendrogenesis in the dentate gyrus and reduction in microglial activity in the dentate gyrus and mPFC in females and males, demonstrating a role for progenitor cells in the dentate gyrus in dependence-induced endothelial and microglial dysfunction. These data suggest that progenitor cells born during withdrawal and abstinence from CIE in the dentate gyrus are aberrant and could play a role in strengthening ethanol memories triggered by ethanol cues via central and peripheral immune responses.

## 1. Introduction

Results from the 2017 National Survey on Drug Use and Health show that moderate to severe alcohol use disorder (AUD) annually affects about 17 million adults in the United States [1]. Relapse, defined as the resumption of alcohol drinking following a prolonged period of abstinence, represents a prevalent and significant public health concern in AUD. Relapse is a major impediment to treatment efforts. One promising approach for reducing the high propensity for relapse in subjects with moderate to severe AUD is the identification of vulnerability factors in the brain that contribute to enhanced relapse to ethanol drinking; the medial prefrontal cortex (mPFC) and hippocampus are the key brain regions implicated in relapse to ethanol drinking behaviors [2]. AUD is associated with neurological deficits, including, but not limited to, loss of brain volume and cognitive impairments, particularly, in functions dependent on the frontal cortex and the hippocampus [3,4,5,6,7,8,9]. Several widely accepted rodent models of alcohol dependence also implicate the structural and functional impairments of the mPFC and hippocampus as neurological factors that contribute to alcoholism [10,11,12,13]. One such model, namely the chronic intermittent ethanol vapor exposure (CIE) model, implements daily cycles of intoxication via ethanol vapors and withdrawal to induce clinical signs of alcoholism, such as somatic withdrawal symptoms and escalated ethanol drinking in rats [14,15]. Impairments in the function of the mPFC and hippocampus produced by CIE could be associated with unregulated drinking patterns observed in CIE animals [16,17,18,19,20,21,22,23,24,25]. Although some of the neural mechanisms underlying unregulated drinking driven by CIE in these brain regions are being uncovered [16,17,18,19,20,21,23,25,26,27,28,29,30,31,32,33,34,35,36], the alterations in endothelial, oligodendroglial, and microglial cells due to relapse and reinstatement that underlie the behavioral changes during dependence are not clearly understood.

CIE also affects other forms of plasticity in the hippocampus and mPFC [16,20,23]. For example, in the dentate gyrus (DG) of the hippocampus, the proliferation of and the survival of progenitor cells into granule cell neurons are reduced during drinking in CIE-exposed rats (i.e., alcohol-dependent rats) and in nondependent rats self-administering ethanol when compared with alcohol-naïve controls [16,20]. Similarly, in the mPFC, the proliferation of and the survival of progenitor cells into glial cells are reduced during drinking in CIE-exposed rats, and this effect is not evident in nondependent rats self-administering ethanol when compared with alcohol-naïve controls [20]. Notably, the reduction in the proliferation of progenitor cells in the DG is evident even with prolonged drinking. However, the reduction in these cells in the mPFC is normalized to control levels after prolonged drinking in CIE rats [20]. These findings suggest that under CIE conditions, the regeneration of proliferating progenitor cells in the DG is hampered with continued drinking and is enhanced in the mPFC, perhaps due to the phenotype of the cells or cell-intrinsic factors regulating their proliferation [37].

A few studies have investigated whether withdrawal and abstinence alter chronic ethanol-induced reductions in the number of progenitors in the DG and mPFC [16,23,38]. The suppression of the number of progenitors following CIE exposure was found to be transient in the DG and mPFC, as an increase in the progenitor pool is evident at early withdrawal [16,23], indicating a burst in proliferation. Importantly, cells born during the proliferative burst differentiate into neural progenitors in the DG and glial progenitors in the mPFC during protracted abstinence [23,38]. However, following CIE exposure, the number of differentiated neural progenitors in the DG that mature into granule cell neurons is significantly reduced compared to nondependent drinking or the control alcohol-naïve condition [16,23]. Therefore, in the CIE model, the stability of the progenitor pool during withdrawal and abstinence in the DG is questionable, and their survival and integration into the granule cell layer may be hindered due to a hostile microenvironment in the neurogenic niche [23]. Interestingly, the number of proliferating progenitors or the progenitor pool in the mPFC and DG in CIE animals is unaltered during prolonged abstinence [16,20]; therefore, it is not clear if the progenitor cells in the DG and mPFC born during protracted abstinence play a role in ethanol seeking behavior in CIE rats.

Additional studies have investigated the role of the progenitor cells born during abstinence in the adult brain in affecting relapse and reinstatement of drug-seeking behaviors [39,40,41,42]; however, none have investigated this role in the context of alcohol dependence. We, therefore, tested the hypothesis that progenitor cells born during forced abstinence from CIE are aberrant and contribute to alcohol-seeking behaviors. We used a pharmacogenetic rat model [43] in which actively dividing progenitor cells can be selectively and inducibly ablated. Our data demonstrate that progenitor cells born during abstinence in CIE female and male rats contribute to the reinstatement of ethanol seeking, and inhibiting this process assisted with reduced seeking when triggered by ethanol cues. More notably, reductions in the progenitor pool in the DG prevented CIE-induced increases in peripheral immune responses, reductions in blood–brain barrier (BBB) tight junction (TJ) proteins, and the activation of microglial cells, suggesting a role for endothelial and microglial dysfunction in ethanol-seeking behaviors via increased progenitor cells.

## 2. Results

### 2.1. Female Rats Consume More Ethanol than Male Rats Prior to and during CIE

**Figure 1:**
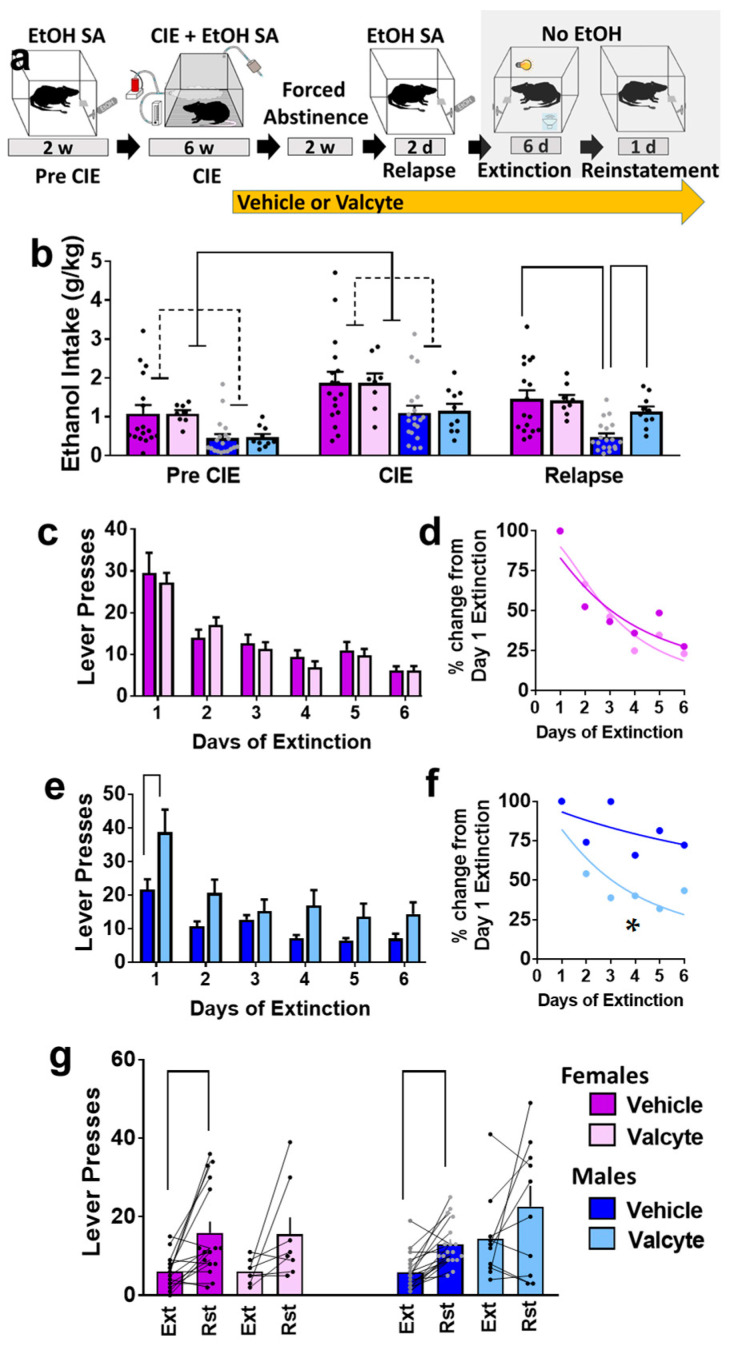
(**a**) Schematic of experimental timeline and experimental group information. All animals were euthanized 45–60 min after reinstatement session. EtOH, ethanol; w, weeks; d, days. (**b**) Amount of ethanol consumed by animals during each stage (pre-CIE is average of last 3 sessions; CIE is average drinking during weeks 4–6; relapse is average of 2 sessions). (**c**,**d**) Responding during extinction sessions is indicated as active lever responses (**c**) or latency to extinguish behavior (**d**) in female rats. (**e**,**f**) Responding during extinction sessions is indicated as active lever responses (**e**) or latency to extinguish behavior (**f**) in male rats. Significance is indicated with lines in (**e**) and asterisk in (**f**). (**g**) Responding during reinstatement of ethanol seeking is presented as active lever presses. Significance is indicated with lines. Lines with heads indicate overall ANOVA. Lines without heads indicate differences with post hoc. Data are expressed as mean ± S.E.M., and significance is set at *p* < 0.05.

Ethanol (10% *v*/*v*) consumption was determined in female and male rats prior to the onset of vapor exposure and during vapor exposure weeks (Figure 1a,b). Repeated measures via two-way ANOVA did not show a sex × CIE interaction (F (1, 50) = 0.12; *p* = 0.9) but showed the main effect of sex (F (1, 50) = 5.0; *p* < 0.01) and the main effect of CIE (F (1, 50) = 40; *p* < 0.001). These data indicate that females consumed more ethanol than males, and both sexes increased ethanol consumption during CIE. In addition, we report rats that were given valcyte or vehicle did not differ in ethanol consumption prior to the onset of vapor exposure and during vapor exposure weeks, indicating a non-biased separation of TK rats into each treatment condition (*p* > 0.05; Figure 1b). Active and inactive lever responses prior to the onset of vapor exposure and during vapor exposure weeks are reported in Supplementary Figure S1a,b.

The vapor exposure during CIE produced the desired range as indicated by their BALs. We report the BALs (mean ± SEM) in weeks 3–6 in mg% (females: 170 ± 17, 180 ± 44, 190 ± 3, 185 ± 15; males: 121 ± 29, 129 ± 13, 152 ± 20, 168 ± 21). Two-way ANOVA revealed a main effect of sex ((F (1, 204) = 6.0; *p* = 0.02) with females having higher BALs compared to males.

### 2.2. Valcyte Enhanced Ethanol Consumption during Relapse Sessions in Male Rats

Rats were treated with valcyte or vehicle during the last week of CIE and were continued on the diet until the end of this study. Two-way ANOVA of ethanol consumed during relapse revealed a sex × valcyte interaction (F (1, 50) = 4.0; *p* = 0.05) and a main effect of sex (F (1, 50) = 12.7; *p* < 0.001) without a main effect of valcyte (F (1, 50) = 2.9; *p* = 0.09). The post hoc analysis revealed higher ethanol consumption in vehicle females compared with vehicle males, and higher consumption in valcyte males compared with vehicle males (*p* < 0.05; Figure 1b). Active and inactive lever responses during relapse sessions in female and male rats are reported in Supplementary Figure S1a,b.

### 2.3. Valcyte Enhanced the Latency to Extinguish Ethanol Responses in Male Rats

Vehicle and valcyte rats experienced extinction sessions over six days. Data from females and males were analyzed separately. In females, repeated measures using two-way ANOVA detected a main effect of days on active lever responses (F (5, 115) = 25.9; *p* < 0.001) but did not reveal a day × valcyte interaction or main effect of valcyte (Figure 1c). Further analysis of active lever responses did not detect differences in the latency to extinguish ethanol-cued responses between vehicle and valcyte groups (Kolmogorov–Smirnov D = 0.33, *p* = 0.9; Figure 1d).

In males, repeated measures via two-way ANOVA detected a day × valcyte interaction (F (5, 130) = 2.5; *p* = 0.03), a main effect of valcyte (F (1, 26) = 9.2; *p* = 0.005), and a main effect of days on active lever response (F (5, 130) = 24.8; *p* < 0.001) (Figure 1e). The post hoc analysis revealed higher active lever responses in the valcyte group compared with vehicle group on the first day of extinction. Further analysis of lever responses also detected differences in the latency to extinguish ethanol-cued responses between vehicle and valcyte groups (Kolmogorov–Smirnov D = 0.83, *p* = 0.02; Figure 1f), in which the valcyte group exhibited an enhanced latency to extinguish compared with the vehicle group. Inactive lever responses did not differ in females and males (Supplementary Figure S1c,d).

### 2.4. Valcyte Reduced Reinstatement of Ethanol-Seeking in Female and Male Rats

Vehicle and valcyte rats experienced reinstatement of ethanol seeking triggered by ethanol context and cues. Data from females and males were analyzed separately. Paired *t* test revealed significant reinstatement compared with the last day of extinction in vehicle-treated female (t(df) = 3.5; *p* = 0.002) and male rats (t(df) = 5.0; *p* < 0.0001). The paired analysis did not reveal significant reinstatement in valcyte-treated female (t(df) = 2.0; *p* = 0.08) and male (t(df) = 1.5; *p* = 0.14) rats (Figure 1g). Inactive lever responses did not differ between extinction and reinstatement sessions in females and males (Supplementary Figure S1e).

### 2.5. Valcyte Significantly Reduces the Number of Actively Dividing Progenitor Cells in the LV and SGZ in Female and Male Rats

**Figure 2:**
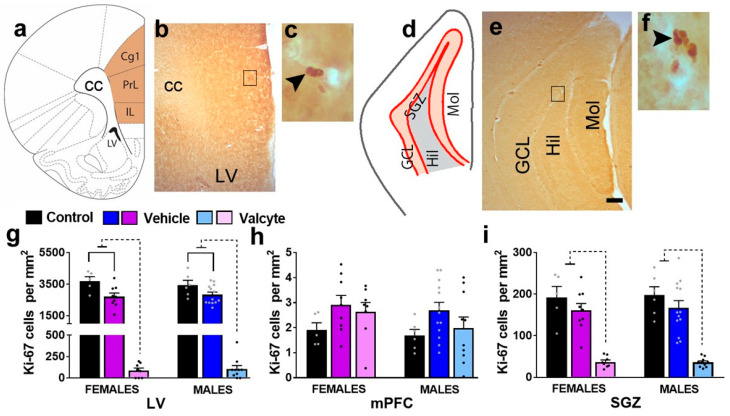
(**a**–**f**) Schematic overview of region of interest in the cortex ((**a**), bregma 2.7) and hippocampus ((**d**), bregma −3.6). Photomicrographs of Ki-67-labeled cells in the mPFC and LV (**b**,**c**) and the granule cell layer (GCL) of the hippocampus (**e**,**f**). Scale bar in (**e**) is 150 μm, which applies to (**b**,**e**). (**g**–**i**) Quantitative analysis of Ki-67-labeled cells in the LV (**g**), mPFC (**h**), and SGZ (**i**). Significant differences are indicated by solid and dashed lines. Data are expressed as mean ± S.E.M.

The effects of vehicle and valcyte treatment on the number of Ki-67-labeled cells were determined via stereological analysis. Ki-67 was used as it is an endogenous marker specific for proliferation [44] and labels all actively dividing cells. The number of cells was quantified in 3 areas of the brain: the subventricular zone of the lateral ventricle (LV), the medial prefrontal cortex (mPFC), and the subgranular zone (SGZ) of the DG (Figure 2a–f).

In the LV, one-way ANOVA detected significant alterations in the number of Ki-67 cells in female (F (2, 19) = 75.4; *p* < 0.001) and male (F (2, 26) = 106; *p* < 0.001) rats (Figure 2g). In female rats, the post hoc analysis revealed that vehicle treatment in CIE rats produced a significant reduction in the number of Ki-67 cells compared with alcohol-naïve controls (*p* = 0.002), and valcyte treatment almost abolished the number of Ki-67 cells compared to naïve controls and vehicle rats (*p* < 0.0001). In male rats, the post hoc analysis revealed that vehicle treatment in CIE rats produced a significant reduction in the number of Ki-67 cells compared with alcohol-naïve controls (*p* = 0.003), and valcyte treatment almost abolished the number of Ki-67 cells compared to naïve controls and vehicle rats (*p* < 0.0001). In the mPFC, one-way ANOVA did not detect any changes in the number of Ki-67 cells in female and male rats (*p* > 0.05; Figure 2h).

In the SGZ, one-way ANOVA detected significant alterations in the number of Ki-67 cells in female (F (2, 19) = 26.2; *p* < 0.001) and male (F (2, 26) = 28.5; *p* < 0.001) rats (Figure 2i). In female rats, the post hoc analysis revealed that vehicle treatment in CIE rats did not alter the number of Ki-67 cells compared with alcohol-naïve controls (*p* = 0.2), and valcyte treatment almost abolished the number of Ki-67 cells compared to naïve controls and vehicle rats (*p* < 0.0001). In male rats, the post hoc analysis revealed that vehicle treatment in CIE rats did not alter the number of Ki-67 cells compared with alcohol-naïve controls (*p* = 0.2), and valcyte treatment almost abolished the number of Ki-67 cells compared to naïve controls and vehicle rats (*p* < 0.0001).

### 2.6. Distinct Peripheral Immune Responses in Female and Male Rats Are Seen after Reinstatement of Ethanol Seeking; Valcyte Prevents These Responses

Differences in the amount of pro-inflammatory (IFN-γ; TNF-α; IL-6), anti-inflammatory cytokines (IL-4; IL-13), and chemokines (CxCL-1) were determined in plasma samples from control, vehicle, and valcyte animals within each sex.

**Figure 3:**
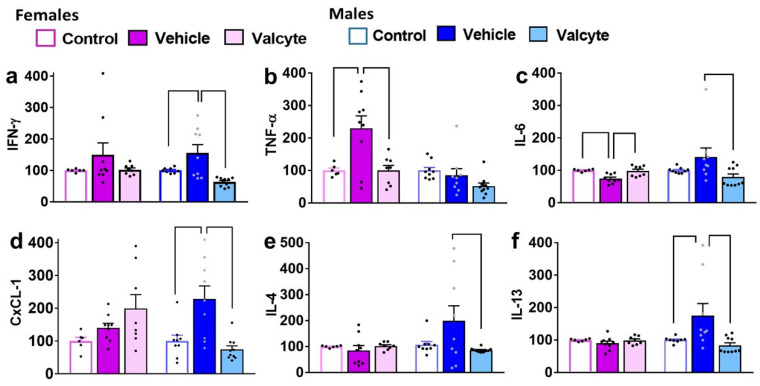
Levels of pro- and anti-inflammatory markers in terminal plasma. Cytokines (**a**–**c**,**e**,**f**) and chemokines (**d**) were measured in the plasma of female and male rats that were controls or CIE experienced with vehicle or valcyte. One-way ANOVA was used to detect differences within each sex. Differences between groups within each sex (*p* < 0.05) are indicated by separate solid lines. Data in treatment groups in each sex are expressed as percent change from controls within each sex and shown as mean ± S.E.M.

In females, IFN-γ was not different between groups. In males, one-way ANOVA detected significant alterations (F (2, 24) = 9.4; *p* < 0.001), with the vehicle group having higher expressions compared to the control and valcyte groups (*p* < 0.05; Figure 3a).

In males, TNF-α was not different between groups. In females, one-way ANOVA detected significant alterations (F (2, 19) = 7.8; *p* = 0.003), with the vehicle group having higher expression compared to control and valcyte groups (*p* < 0.05; Figure 3b).

In females, one-way ANOVA detected significant alterations in IL-6 levels (F (2, 19) = 9.1; *p* = 0.001), with the vehicle group having lower expressions compared to control and valcyte groups (*p* < 0.05; Figure 3c). In males, one-way ANOVA detected significant alterations (F (2, 24) = 3.6; *p* = 0.03), with the vehicle group having higher expressions compared to the valcyte group (*p* < 0.05; Figure 3c).

In females, CxCL-1 was not different between groups. In males, one-way ANOVA detected significant alterations (F (2, 24) = 10.6; *p* < 0.001), with the vehicle group having higher expressions compared to the control and valcyte groups (*p* < 0.05; Figure 3d).

In females, IL-4 was not different between groups. In males, one-way ANOVA detected significant alterations (F (2, 24) = 3.3; *p* = 0.05), with the vehicle group having higher expressions compared to the valcyte group (*p* = 0.05; Figure 3e).

In females, IL-13 was not different between groups. In males, one-way ANOVA detected significant alterations (F (2, 24) = 5.3; *p* = 0.01), with the vehicle group having higher expressions compared to the control and valcyte groups (*p* < 0.05; Figure 3f).

In females and males, IL-10 was not different between groups.

### 2.7. Valcyte Prevents Abstinence-Induced Reduction in Endothelial Tight Junction Protein cld5 in the DG in Female and Male Rats

The effects of vehicle and valcyte treatment in male and female CIE rats on the density of proteins associated with neuroimmune responses (Cox-2, NF-κB) and blood–brain barrier integrity (adherens junction protein Cdh5 and tight junction protein Cld5) were investigated.

**Figure 4:**
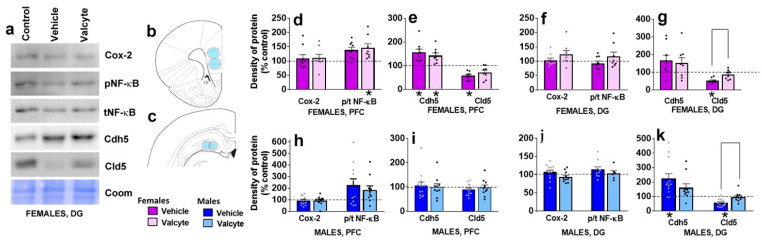
Western blotting analysis of tight junction proteins and neuroinflammatory markers in the prefrontal cortex (PFC, (**b**), bregma 2.7) and dentate gyrus ((**c**), bregma −3.6) of female and male rats that were in control, vehicle or valcyte groups. Colored circles in coronal brain section images (**b**,**c**) indicate area of tissue punches collected for lysis. Representative immunoblots with corresponding coomassie (Coom) blot are indicated in (**a**). Cyclooxygenase-2, Cox-2; Claudin-5, Cld5; VE-Cadherin, Cdh5; Nuclear Factor κ B, NF-κB. (**d**–**k**) Quantitative data represented as mean ± S.E.M. of percent change in density of protein from respective controls (indicated as dashed line). Differences between vehicle and valcyte groups within each sex (*p* < 0.05) are indicated with separate solid lines. Differences between control and treatment are indicated with an asterisk symbol. Data are expressed as mean ± S.E.M.

In the mPFC, vehicle or valcyte treatment did not alter the expression of Cox-2 in female and male rats (Figure 4d,h). We next determined the ratio of phosphorylated to total NF-κB expressions to evaluate the effects of treatments on the activity state of the protein. Valcyte treatment significantly enhanced the expression of p/t NF-κB in females (F (2, 19) = 4.2; *p* < 0.05), and this effect was not evident in males (Figure 4d,h). Vehicle and valcyte treatment significantly enhanced the expression of Cdh5 in females (F (2, 19) = 8.2; *p* < 0.05; Figure 4e,i), and this was not evident in males. Vehicle treatment reduced the expression of Cld5 in females (F (2, 19) = 5.9; *p* < 0.05) and this was not prevented with valcyte (Figure 4e,i). Cld5 was not altered in males.

In the DG, vehicle or valcyte treatment did not alter the expression of Cox-2 or p/t NF-κB in female and male rats (Figure 4f,j). Vehicle treatment significantly enhanced the expression of Cdh5 in males (F (2, 26) = 3.6; *p* < 0.05), and this was not evident in females (Figure 4g,k). Vehicle treatment reduced the expression of Cld5 in females (F (2, 19) = 11.5; *p* < 0.05) and males (F (2, 26) = 8.0; *p* < 0.05), and this was prevented with valcyte in both sexes (*p* < 0.05; Figure 4g,k).

### 2.8. Valcyte Prevents Abstinence-Induced Activation of Microglial Cells in the mPFC and DG in Female and Male Rats

We evaluated the state of Iba-1-labeled microglia (total processes length, area of cell soma) in female and male CIE rats under vehicle and valcyte conditions, as these morphological characteristics define the activity state of these cells [45].

**Figure 5:**
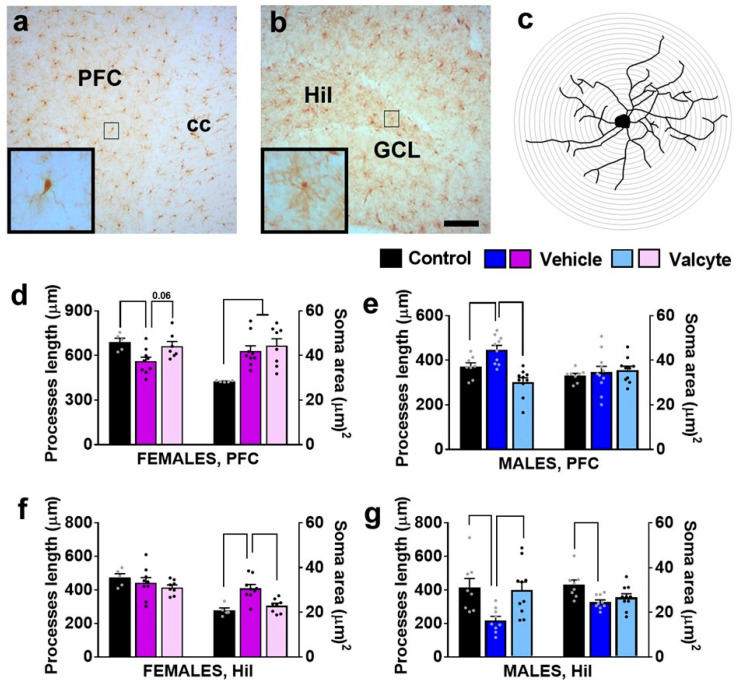
Photomicrographs of Iba-1-labeled cells in the mPFC (**a**) and dentate gyrus (**b**) with examples of individual cells in insets zoomed in from the box in the main panel from one control rat. (**c**) Example of traced Iba-1 cell with sholl ring indicating branches of processes. Each ring is 1 μm apart. Scale bar in (**b**) is 100 μm, which applies (**a**,**b**) main panel. (**d**–**g**) Morphological analysis of Iba-1-labeled cells in the mPFC (**d**,**e**) and hilus (Hil) of the hippocampus (**f**,**g**). Differences between groups within each sex (*p* < 0.05) are indicated with separate solid lines. Data are expressed as mean ± S.E.M.

In the mPFC, there was a significant effect of treatments on total process lengths in females (F (2, 18) = 5.3; *p* = 0.01) and males (F (2, 28) = 15.3; *p* < 0.001). The post hoc analysis revealed that vehicle treatment reduced total process lengths in females and increased length in males (*p* < 0.05). Notably, valcyte treatment prevented these effects in both sexes (strong trend *p* = 0.06 in females and *p* < 0.05 in males; Figure 5d,e). A significant effect of treatments on cell soma area was seen in females (F (2, 18) = 8.5; *p* = 0.002) and was not evident in males. The post hoc analysis in females showed that cell soma area was increased with vehicle and valcyte treatments (*p* < 0.05).

In the hilus of the DG, there was a significant effect of treatments on cell soma area in females (F (2, 18) = 12.3; *p* = 0.004) and in males (F (2, 28) = 5.3; *p* = 0.01). Post hoc analysis in females showed that cell soma area was increased with the vehicle, and valcyte treatment prevented these effects (*p* < 0.05; Figure 5f). Post hoc analysis in males showed that cell soma area was reduced with the vehicle, and valcyte treatment prevented these effects (*p* < 0.05; Figure 5g). A significant effect of treatments on total process lengths was evident in males (F (2, 28) = 6.2; *p* = 0.006) and not in females. Post hoc analysis revealed that vehicle treatment reduced total process lengths and valcyte treatment prevented these effects (*p* < 0.05; Figure 5g).

### 2.9. Valcyte Enhances the Number of Oligodendrocytes in the DG in Male Rats

**Figure 6:**
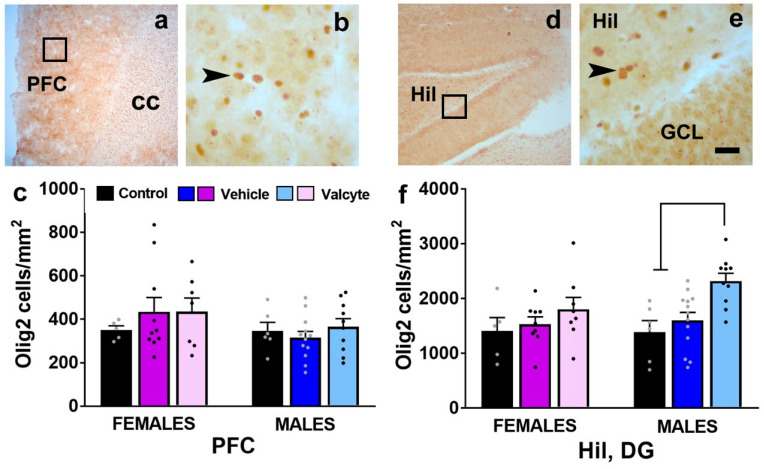
(**a**,**b**,**d**,**e**) Photomicrographs of Olig2 stained brain sections of the mPFC (**a**,**b**) and hippocampus (**d**,**e**) from one control rat. Boxes in (**a**,**d**) indicate the region of cells magnified in (**b**,**e**). Arrowhead in (**b**,**e**) point to Olig2 stained cell. Scale bar in (**e**) is 20 μm, which applies to (**b**,**e**). (**c**,**f**) Quantitative analysis of Olig2-labeled cells in each treatment group in each sex in the PFC and the hilus (Hil) of the dentate gyrus of the hippocampus. Values are mean ± S.E.M. Differences between groups within each sex (*p* < 0.05) are indicated with separate solid lines.

We also determined the number of Olig2 cells in the mPFC and the hilus of the DG as CIE and abstinence from CIE significantly alter the expression of markers associated with oligodendrogenesis and myelination [46]. The PFC one-way ANOVA did not detect a significant change in the number of cells between groups in female and male rats (Figure 6c). In the hilus of the DG, one-way ANOVA did not detect any differences between groups in female rats but detected significant alterations in the number of Olig2 cells in male rats (F (2, 26) = 8.5; *p* < 0.001). Post hoc analysis revealed a higher number of Olig2 cells in valcyte-treated rats compared with alcohol-naïve and vehicle-treated rats (*p* < 0.05; Figure 6f).

## 3. Discussion

The CIE model of alcohol dependence is useful in modeling moderate to severe alcohol use disorder in humans. The present results in GFAP-TK CIE rats are the first to show that hippocampal progenitor cells born during abstinence from chronic ethanol experience play a direct role in the reinstatement of ethanol-seeking behavior in females and males. Mechanisms underlying the progenitor cell-induced reinstatement of ethanol seeking include the activation of immune responses, both centrally and peripherally, and the reduction in tight junction proteins that assist with BBB integrity, specifically in the DG. These findings help demonstrate that the proliferation of progenitor cells during abstinence in the DG in ethanol-dependent female and male rats is aberrant and contributes to ethanol memories that assist with the reinstatement of ethanol-seeking behaviors.

Few studies have attempted to investigate the role of the hippocampus in the context-driven reinstatement of ethanol-seeking behaviors. Correlative studies demonstrate that the reinstatement of ethanol seeking driven by the ethanol context is accompanied by increases in the transcription and protein synthesis of markers of neuronal activation in the hippocampus [47,48,49,50]. More notable is that the pharmacological inactivation of the hippocampus prevents the reinstatement of ethanol seeking triggered by ethanol context, indicating a direct role of the hippocampus in ethanol-context memories and motivation to seek ethanol [50]. Furthermore, ethanol seeking in ethanol-dependent animals could be resulting from neuroplastic and neuroadaptive changes in the DG associated with adult hippocampal neurogenesis (reviewed in [24,51,52,53,54,55,56]). Such neuroplastic changes could involve the proliferative burst in the progenitor cells in the DG 72 h into withdrawal from CIE, a time frame associated with negative affect [16,23]. The effects these progenitor cells have on ethanol seeking remain unclear and were determined in this current study. In addition, the mechanisms by which they regulate seeking were explored.

Male and female GFAP-TK rats with intact progenitors self-administered ethanol and experienced CIE for 6 weeks, following which they were withdrawn from CIE. Female rats showed higher drinking compared with males prior to CIE and during CIE. The higher ethanol drinking in female rats supports the many previous publications on sex differences in ethanol intake in rodent models [57] but appears to be somewhat different from that reported in the human condition, where men consume alcohol at higher levels compared with women [58,59]. It is notable that the clinical research on the transition to alcohol dependence report that once women begin drinking regularly, they progress faster than men to drinking-related problems [60,61,62], supporting the clinical significance of the increases in ethanol drinking in female rats under CIE conditions compared with males.

We used GFAP-TK rats in which neural progenitors can be selectively and inducibly ablated [39,43]. Valcyte was administered during the last week of CIE and was continued into abstinence until the end of this study. Valcyte did not alter relapse to ethanol seeking in female rats, suggesting that newly born progenitors did not influence or support plasticity that assisted with relapse to ethanol seeking. Valcyte, however, enhanced relapse to ethanol seeking in males, indicating that newly born progenitors during abstinence were supporting plasticity to reduce drinking during relapse. The sexually dimorphic effects of valcyte on relapse to drinking may be due to distinct responses in a proliferative burst of neural progenitor cells in the DG in females versus males during early abstinence from CIE, with males demonstrating a rebound burst in proliferating neural progenitors [16,23] and females not showing this effect [63]. It is also possible that an intact progenitor pool in males during abstinence assists with forgetting ethanol memories and reducing negative affect to maintain lower relapse to ethanol drinking compared with females [40,41,42,64,65]. This hypothesis needs to be tested.

Opposite to the above hypothesis, valcyte enhanced latency to extinguish operant responses for ethanol drinking in male rats without any effects on extinction responses in females. Notably, valcyte prevented the reinstatement of ethanol seeking triggered by ethanol cues and contexts in males and females. These findings indicate that progenitors born during abstinence assist with plasticity that prevents extinction learning and, therefore, could assist with facilitating the reinstatement of ethanol-seeking behaviors [39,63]. Taken together, the data suggest that exposure to CIE may create a higher vulnerability for hippocampal impairment in male subjects when compared with female subjects. Additionally, the findings support that newly born progenitors during abstinence in females and males play a mechanistic role in the motivation to seek ethanol.

Male and female rats that received valcyte showed a reduction in progenitor cells in the subventricular zone (SVZ) and DG indicating that valcyte ablated the proliferation of progenitors in these regions. The progenitor pool in the SVZ has stem cells with greater pluripotency compared with the cells in the DG [66,67], and these cells differentiate and migrate to the olfactory bulb through the rostral migratory stream to generate interneurons in the olfactory bulb [68]. Previous observations in the SVZ have indicated that CIE suppressed proliferation and neurogenesis but spared a population of Sox-2-expressing stem cells. Notably, withdrawal from CIE produced rebound increases in proliferation and neurogenesis, and abstinence reduced the effect, suggesting that these cells could play a role in the reinstatement of ethanol-seeking behaviors [16]. Interestingly, human subjects with AUD experience impairment in smell sensation (hyposmia). However, SVZ proliferation and neurogenesis were unaffected [69]. Therefore, it is likely that the reduction in proliferating cells in the SVZ in CIE rats could be resulting from alcohol-induced toxicity and may not contribute to motivational behaviors impaired in alcoholism. Unlike the SVZ, in the DG, human subjects with AUD show reduced levels of progenitor cells and neurogenesis [70,71], supporting the various animal studies that detect similar changes in models of moderate to severe AUD. Therefore, the inhibitory effect of ethanol on the regenerative capacity of the adult hippocampus is considered a precursor for ethanol-induced neurodegeneration in the hippocampus [54,55]. Furthermore, it appears that the withdrawal and early abstinence-induced rebound effect in progenitor cells in the DG is associated with hyperactivity stemming from the neurocircuitry underlying the ethanol withdrawal-induced kindling-like behaviors [24]. These behaviors cause a hyperglutamatergic state and produce hippocampal excitotoxicity, which may be decisive factors for the maintenance of dependence in the long term [72,73,74,75,76]. Supporting this, our findings indicate that progenitors in the DG born during withdrawal and abstinence play a role in the reinstatement of ethanol seeking triggered by ethanol context and cues and, therefore, contribute to the motivational behaviors impaired in moderate to severe AUD. However, the cellular mechanisms regulating the ethanol-withdrawal-induced generation of progenitors in the DG have not been identified [21,23,56], and future mechanistic studies are needed to address the issue.

We explored whether valcyte altered levels of circulating inflammatory markers. This is because studies from cellular models, animal models, and human samples indicate that acute and chronic alcohol exposure interferes with cellular immune responses [77,78,79]. In fact, pro-inflammatory agents, including cytokines and chemokines, have emerged as vital conduits of ethanol’s effects on the central nervous system, and mechanistic studies demonstrate that these agents govern the progression of ethanol-induced pathology, as well as influence the development of AUD. For example, findings from human studies show that low amounts of alcohol drinking decrease circulating plasma cytokine levels, whereas high binge drinking increases plasma cytokine levels [80,81]. The significant immune responses in individuals engaging in alcohol abuse are evident as reduced granulocyte and lymphocyte production, bone marrow suppression, diminished bactericidal activity, and associated infection-related morbidity and mortality [79]. In support of these immune changes, the postmortem brain tissue of individuals with moderate to severe AUD shows increased levels of chemokines and pro-inflammatory cytokines [82,83]. In support of the clinical findings, preclinical studies using a variety of ethanol exposure paradigms indicate immune responses in the brain and serum and that the pro-inflammatory cytokines IL-6, IL-1β, IL-10, and TNF-α, are amongst the most well-described targets of ethanol’s action on immune signaling in the brain [84,85,86,87,88,89,90,91,92,93]. However, the literature concerning the functional effects of cytokines on ethanol-related behaviors is comparatively scant, though the behavioral validation of the changes in neuroimmune-associated genes has emerged. For instance, IL-6 and IL-10, amongst other neuroimmune factors, have been shown to have effects on ethanol consumption and, to a lesser extent, some intoxication-related behaviors [94,95,96]. Our findings show that valcyte reversed levels of circulating inflammatory responses in females and males with distinct profiles. For example, in females, the reinstatement of ethanol seeking was associated with increases in pro-inflammatory cytokines TNF-α and a reduction in IL-6, and valcyte reversed these effects. In males, the reinstatement of ethanol seeking was associated with increases in pro-inflammatory cytokines IFN-γ, IL-6, chemokine CxCL-1, and anti-inflammatory cytokines IL-4 and IL-13, all of which were reversed with valcyte. These sex-specific effects of the reinstatement of ethanol seeking on peripheral immune activity add to the preclinical studies that have indicated higher activity in females compared to males under healthy control conditions [97,98,99] and to the limited data on the effect of ethanol dependence on circulating plasma in both sexes [89,92]. Notably, our findings indicate that progenitor cells play a role in regulating the levels of circulating immunogens in the context of alcohol dependence. Thus, changes in cytokine expression can be a biomarker for the effects of alcohol, though further work is necessary to fully understand their effects in AUD.

Regarding non-neuronal correlates, our initial studies showed reactive oligodendrogenesis in the mPFC during protracted abstinence following CIE-induced dependence in both sexes [23,100,101,102]. The reactive oligodendrogenesis was associated with enhanced expression of the endothelial cell-specific angiogenesis marker PECAM-1 in the mPFC in both sexes [101,102]. Treatment with the anti-angiogenic drug endostatin reduced PECAM-1 expression in both sexes but reduced relapse to drinking in female rats without affecting drinking in males and, in parallel, prevented the neuronal adaptations associated with the relapse of ethanol drinking in females [102]. In this current study, valcyte did not affect the number of progenitors in the mPFC, suggesting that progenitors in this region did not play a role in the reinstatement of ethanol seeking behaviors. However, it is possible that valcyte prevented the withdrawal-induced hyperproliferation in the mPFC to inhibit certain forms of glial plasticity, such as microglial activity to reduce reinstatement of ethanol seeking.

We next examined the mechanisms associated with reduced progenitor cells in the DG and focused on neuroimmune responses. We explored the proteins critical for BBB integrity and the activity of microglial cells and oligodendrocytes in the DG. This is because alterations in the composition of the BBB (via the reduced expression of tight junction (TJ) and adherens junction (AJ) proteins or enhanced expression of endothelial cell adhesion molecules in endothelial cells) are assisted by inflammatory mediators such as cytokines [103,104,105,106]; the enhanced endothelial response also promotes leukocyte emigration via BBB disruption [107,108]. Since TJ and AJ form endothelial cell–cell junctional complexes to restrict the entry of proteins and inflammatory cells into the brain to protect the brain microenvironment, and acute ethanol ex vivo reduces the expression of these proteins, it is possible that ethanol in vivo also produces such alterations in endothelial junction proteins to disrupt BBB integrity and induce neuroinflammation. Given the recent evidence that neuroinflammation plays a significant role in dependence-induced drinking in CIE animals [35,92,109], we examined whether endothelial damage (via reduced expression of TJ and AJ proteins) in the DG occurred coincidentally with the reinstatement of ethanol seeking. Our findings show that the AJ protein Cdh5 was not regulated by valcyte in the DG. However, our results show that valcyte rescued effects in TJ protein Cld5 in the DG in both sexes and suggest that the expression of Cld5 could be compromised by the enhanced circulating immune factors in vehicle rats [110]. We also examined alterations in the levels of NF-κB, a transcription factor that regulates the expression of cytokines, and Cox-2, a key mediator of inflammatory responses [111]. The reinstatement of ethanol seeking did not produce any significant alterations in the levels of these proteins, and valcyte did not affect their expressions. Together, our data suggest that newly born progenitors played a role in motivational aspects of ethanol seeking via alterations in BBB integrity. A potential limitation in the interpretation of these findings is that additional markers of BBB integrity need to be assessed to confirm the effects of ethanol and newly born progenitors on BBB dysfunction.

We also determined the morphological changes in Iba-1 expressing microglial cells to evaluate the activity state of these cells in the hilus of the DG in both sexes, as these morphological characteristics define the activity state of these cells [45]. The reinstatement of ethanol seeking in females and males was associated with morphological changes that reveal the activated state of Iba-1-labeled microglia (altered processes length and soma size), in the DG. These alterations were ameliorated with valcyte and suggest that progenitors born during abstinence could have played a role in the activity of microglial cells to promote the reinstatement of ethanol seeking [112]. Lastly, we determined whether the reinstatement of ethanol seeking was associated with alterations in levels of oligodendrocytes in the DG, a cell type that responds to microglial activation and circulating chemokines and cytokines that modulate immune responses [113]. Our findings do not show any alterations in the number of oligodendrocytes under vehicle conditions but reveal an enhanced number of oligodendrocytes with valcyte, specifically in males. Taken together, the cellular alterations observed as a consequence of the reinstatement of ethanol seeking in the hippocampus may result from progenitor cells generated during abstinence. Although we do not provide a direct link to demonstrate that hippocampal progenitors are a vulnerability factor for ethanol seeking following extinction, we demonstrate that the altered plasticity in the DG in the context of immune responses may contribute to motivational behaviors assisting with the reinstatement of ethanol seeking.

## 4. Materials and Methods

### 4.1. Animals

Transgenic rats expressing herpes simplex virus-thymidine kinase HSV-TK under the human GFAP promoter (GFAP-TK) were generated on a Long–Evans background [43]. These rats were bred at the VA San Diego Healthcare System. Rats were weaned at 21–24 d of age, pair housed, and genotyped via qPCR. Two to three rats were housed per cage in a temperature-controlled (22 °C) vivarium on a 12 h/12 h light/dark cycle (lights on 9:00 P.M.–9:00 A.M.). All procedures were performed during the dark phase of the light/dark cycle. Food and water access was available ad libitum. All rats weighed approximately 180–250 g and were 8 weeks old at the beginning of this study. All experimental procedures were carried out in strict adherence to the National Institutes of Health Guide for the Care and Use of Laboratory Animals (NIH publication number 85–23, revised 1996) and approved by the Institutional Animal Care and Use Committee of VA San Diego healthcare system. Sixty-eight adult GFAP-TK rats completed this study.

### 4.2. Ethanol Self-Administration

The behavioral experiments conducted herein are presented as a detailed schematic in Figure 1a. Twenty-five female and twenty-eight male experimentally naïve rats were given one 4 h lever-responding training session in the operant conditioning boxes (Med Associates Inc., Fairfax, VT, USA) on a fixed-ratio 1 schedule (FR1; one response resulted in one reinforce delivery), where one press on the available lever resulted in the delivery 0.1 mL of water to a sipper cup mounted on the wall in between the two levers. The operant conditioning boxes were housed inside sound-attenuating chambers. During these sessions, the house light and white noise were turned off, and lever responses were paired with a cue light. Subsequently, the rats were trained to discriminate between two available levers to obtain 0.1 mL ethanol (10% *v*/*v*) [14,113,114]. All other conditions remained the same as before. However, one lever (right, active lever) was paired with a cue light, and lever responding resulted in the delivery of ethanol, while responding on the inactive (left) lever was recorded but had no programmed consequence. Each ethanol delivery was followed by a 4 s time-out during which response to the active lever did not result in the delivery of ethanol. During this time-out period, the cue light above the active lever remained on; thus, the cue light was paired with the delivery of ethanol. Rats had two days of 2 h FR1 sessions followed by daily 30 min sessions. These 30 min sessions continued until stable response was obtained, where stable response was defined as less than 10% variation in active lever response for 3 consecutive 30 min FR1 sessions. Subsequently, the rats received chronic intermittent ethanol vapor exposure (CIE; see procedure below) for a duration of 6 weeks and received two 30 min FR1 ethanol drinking (ED) sessions per week during these 6 weeks. Responding was analyzed to determine the escalation of self-administration compared to pre-vapor stable responses.

### 4.3. Chronic Intermittent Ethanol Vapor Exposure (CIE)

During CIE, rat cages (2 males per cage; 3 females per cage) were housed in specialized chambers and were exposed to alcohol vapors on a 14 h ON/10 h OFF schedule. Alcohol (95% ethanol, *v*/*v*; cat # 4355221, Fisher Scientific, Hampton, NH, USA) from a large reservoir was delivered to a heated flask at a regulated flow rate using a peristaltic pump (model QG-6, FMI Laboratory, Bloomfield, CT, USA). The drops of alcohol in the flask were immediately vaporized and carried to the vapor chambers containing the rat cages by controlled air flow (regulated by a pressure gauge). The air pressure and ethanol flow rates were optimized to obtain blood alcohol levels (BALs) between 125 and 250 mg% (mg/dL) or 27.2 and 54.4 mM [114]; these BALs are 2–3 times the BAL observed in binge drinking, but not high enough to abolish righting reflex [115,116]. Tail bleeding for BALs was performed according to [23].

### 4.4. Suppression of Cell Proliferation

Proliferation of progenitor cells was suppressed by feeding the animals the orally available prodrug, valganciclovir (Valcyte, Roche, Little Falls, NJ, USA), which is enzymatically converted to ganciclovir. Valcyte (7.5 mg) was given in a 0.5 g pellet of a 1:1 mixture of ground chow and almond butter. To minimize neophobia, rats were exposed to the chow–almond butter mixture in their home cage for 2–4 days prior to drug treatment. On drug treatment days, each rat was separated into an empty cage without bedding, and individual Valcyte pellets were placed on the wall of the cage and monitored for feeding activity to ensure consistent dosing. Once the animal consumed the drug pellet, the animal was moved back to the housing chamber. The entire feeding procedure lasted between 4 and 7 min, and care was taken to reduce any stressful experience. Valcyte treatment (1×/d) was initiated during week 6 of CIE (at least 3 h before the self-administration session) and was continued at 3x/week until the day of euthanasia. All GFAP-TK animals consumed the vehicle or Valcyte chow/almond butter pellet (vehicle/valcyte; females, *n* = 17 vehicles, *n* = 8 valcyte; males: *n* = 18 vehicles, *n* = 10 valcyte). Previous report indicates that the ablation of proliferating cells with valcyte is evident 5–7 days after initiation of treatment [43]. Therefore, we assume that the number of proliferating cells in valcyte-treated animals was very few to none during the second week of abstinence and until the end of this study.

### 4.5. Relapse Drinking during Abstinence

After two weeks of abstinence from CIE and ethanol self-administration, vehicle/valcyte CIE rats underwent two 30 min FR1 sessions to lever press for ethanol reinforcement (0.1 mL of 10% *v*/*v* ethanol) under cue-context conditions identical to that used for training and maintenance. Active and inactive lever responses were recorded.

### 4.6. Extinction

Following relapse, vehicle/valcyte CIE rats were subject to 6 daily 30 min extinction sessions under a different cue-context combination than that used for training and maintenance. Specifically, operant boxes different from those used for self-administration were used, the house light and white noise were turned on, and no cue lights were available following lever presses (Figure 1a). Finally, the lever response did not result in the delivery of ethanol. Both lever responses were recorded.

### 4.7. Reinstatement

Following the 6th day of extinction, rats were subject to one 30 min session of cued-context reinstatement of ethanol seeking. Specifically, rats were introduced to operant chambers under conditions identical to training and maintenance (no house light, no white noise; original operant box). Active lever responses resulted in the presentation of the cue light for 4 s but did not result in the delivery of ethanol. Both active and inactive lever responses were recorded. Forty-five minutes to sixty minutes after reinstatement, all animals were euthanized under anesthesia via rapid decapitation.

### 4.8. Terminal Plasma and Brain Tissue Collection

After the reinstatement session, TK CIE rats (vehicle/valcyte; females, *n* = 17 vehicles, *n* = 8 valcyte; males: *n* = 18 vehicles, *n* = 10 valcyte) and time- and age-matched TK ethanol-naïve rats (*n* = 6 females; *n* = 9 males; housed in the Vivarium under similar light cycle conditions with food and water available ad libitum) were euthanized via rapid decapitation.

Trunk blood was collected from all naïve rats and all valcyte-treated rats, and a subset of vehicle-treated rats (females, *n* = 9 vehicle; males: *n* = 9 vehicle) prior to brain tissue collection. Trunk blood was not collected from the other vehicle-treated rats as we had restricted the number of samples for plasma ELISAs due to high cost of sample processing. One ml of blood was collected in heparinized tubes and centrifuged at 4000 rpm for 5 min at 4 °C with supernatant plasma collected for total protein quantification and cytokine analysis. Protein concentration was determined using a detergent-compatible Lowry method (Bio-Rad, Hercules, CA, USA). Samples were stored at −80 °C.

Brains were isolated from a subset of TK naïve rats (*n* = 5 females; *n* = 8 males), all valcyte-treated rats, and all vehicle-treated rats. However, brains for histology/Western blotting were processed for naïve rats and valcyte rats and a subset of vehicle-treated rats (females, *n* = 9 vehicles; males: *n* = 13 vehicles) and dissected along the midsagittal plane. The left hemisphere was snap frozen for Western blotting analysis, and the right hemisphere was postfixed in 4% paraformaldehyde for immunohistochemistry. For tissue fixation, the hemispheres were incubated at room temperature for 36 h and, subsequently, at 4 °C for 48 h with fresh paraformaldehyde replacing the old solution every 12 h. Finally, the hemispheres were transferred to sucrose solution (30% sucrose with 0.1% sodium azide) for cryoprotection and storage until tissue sectioning was conducted [117]. Brains from the remaining vehicle-treated rats were processed for ELISAs and were not used as our protocol did not work out. While all valcyte rats are overlapping for plasma and brain (histology/Western blotting), only one vehicle female and four vehicle males are overlapping for these analyses. The behavior of all animals used in the biochemical (ELISA and brain) analyses is presented. Therefore, not all rats were processed for all biochemical analyses, and the selection was not based on behavioral responses. As a result, we did not correlate any biochemical measures with behavioral responses.

### 4.9. Multiplex Cytokine Immunoassays

Cytokine levels in plasma were analyzed using a custom Meso Scale Discovery rat cytokine v-plex panel, allowing for the measurement of interferons (IFN-γ), adipokines (TNF-α), interleukins (IL-1β, IL-4, IL-5, IL-6, IL-10, IL-13), and chemokines (KC/GRO (CXCL1); (catalog #: K15059D, MSD, Rockville, MD, USA)). Plasma samples were diluted by adding 30 uL plasma to 90 uL of diluent provided with the kit, and 50 uL of the diluted plasma was used per well. All samples were run in duplicates. Plates were read with a Sector Imager 2400, and data were analyzed using the MSD Discovery Workbench software v. 4.0 (MSD, Rockville, MD, USA). The lower limit of detection (LLOD) for the assays varied by analyte. The following are LLOD for each marker (pg/mL): IFN-γ = 0.65, IL-1β = 6.92, IL-4 = 0.69, IL-5 = 14.1, IL-6 = 13.8, IL-10 = 16.4, IL-13 = 1.97, KC/GRO (CXCL1) = 1.04, and TNF-α = 0.72. Values falling below the LLOD were replaced with 0 pg/mL in all analyses. IL-1β and IL-5 were not detected in plasma. Data are represented as percent change from control (ethanol naïve) condition, and samples from each plate were compared to their respective controls. Controls from plate 1 were compared with controls from plates 2–5.

### 4.10. Quantitative Immunohistochemistry Analysis for Ki-67-, Olig2-, and Iba-1-Labeled Cells

For Ki-67 (marker for cellular proliferation, a transcription factor expressed in cells in S, G2, and M phase of cell cycle) and Olig2 (marker for oligodendrocytes, a transcription factor expressed by oligodendrocytes), quantitative immunohistochemical assay performed using a previously published optical fractionator method [101,118]. Ki-67-labeled cells were quantified in the subventricular zone, mPFC, and subgranular zone with a Zeiss AxioImager Microscope equipped with Stereo Investigator (MicroBrightField Bioscience, Williston, VT, USA), a three-axis Mac 5000 motorized stage, a Zeiss digital MRc video camera, PCI color frame grabber, and computer work station. Subventricular zone and mPFC (4 sections per rat, bregma 3.7 to 2.2) and subgranular zone (6 sections per rat, bregma −1.4 to −6.7) regions were contoured, as indicated in Figure 2a–f, using a 2.5× objective with a 10× eyepiece and the above software. Cells were visually quantified within the contour using a 20× objective and a 10× eyepiece by an observer blind to this study using the following criteria: cells stained as dark brown to black, with the ability to focus the boundary of the cell within the mounted section thickness. A software generated 180 × 120 µm counting frame, which was systematically moved through the entire contoured area of the tissue to manually assess and count the positive cells. Mounted section thickness after immunohistochemistry was determined to be ~28 µm. The Ki-67 immunoreactive cells always appeared in clusters of irregularly shaped dark-stained cells (Figure 2b,f). The overlapping-pair arrangement and the number of cells in each cluster were confirmed by focusing on different layers of cells along the Z-axis. Olig2 immunoreactive cells were quantified in the prelimbic cortex of the mPFC (2 sections per rat; bregma 3.7 to 2.2) and the hilus of the DG (2 sections per rat; bregma −1.4 to −6.7). Olig2 cells mostly appeared as individual cells with smooth and rounded cell shapes (Figure 6b,e). Absolute cell counting (complete counting of all immunoreactive cells in the contoured area) was performed; the data are presented as the total number of cells per unit area (cells/mm^2^ based on mounted section thickness) per animal.

Analysis of Iba-1 (protein expressed in macrophages and microglial cells)-labeled microglial cells in the prelimbic cortex of the mPFC and hilus of the DG was performed using a previously published protocol [102]. Zeiss Axiophot AxioImager A2 microscope was used to view the cells, and Neurolucida (MicroBrightField) was used to create three-dimensional (3D) tracings and Sholl analysis (Figure 5a–c). The following criteria were used to access the morphology of Iba-1 cells: (1) the cell was in the prelimbic cortex or the hilus (the region of interest); (2) the cell was specifically defined from other cells; (3) the cell was fully intact and not broken; (4) the cell was stained dark enough to visualize the soma and processes. A total of 2–6 cells were traced for each rat, and 2–3 were traced from each of the two sections using the 40× magnification (equipped with a 10× eyepiece). After tracing, 3D Sholl analysis was performed to determine the total number of process intersections relative to the radial distance (starting from 0 µm and increasing to 1 µm from the soma). Additional cellular characteristics, including cell soma area and total process lengths, were analyzed.

### 4.11. Western Blot Analysis

Tissue punches from 500 um thick sections of mPFC and hippocampus (Figure 4b,c) were homogenized via sonication in ice-cold buffer (320 mM sucrose, 5 mM HEPES, 1 mM EGTA, 1 mM EDTA, 1% SDS, with Protease Inhibitor Cocktail and Phosphatase Inhibitor Cocktails II and III diluted 1:100; Sigma, St. Louis, MO, USA), and protein concentration was determined using a detergent-compatible Lowry method (Bio-Rad, Hercules, CA, USA). Brain tissue lysates (30 μg) were mixed (1:1) with a Laemmli sample buffer containing β-mercaptoethanol. Samples were run on 10% SDS-PAGE gels (Bio-Rad) and transferred to polyvinylidene fluoride membranes (PVDF pore size 0.2 μm). Blots were blocked with 5% milk (*w*/*v*) in TBS (25 mM Tris−HCl (pH 7.4), 150 mM NaCl) for one hour at room temperature and were incubated with the primary antibody for 16–20 h at 4 °C: Antibody to phosphorylated-NFκB-p65 at Ser536 (rabbit polyclonal, 1:200, Cell Signaling cat# 3033L, molecular weight 65 kDa); total NFκB-p65 (rabbit polyclonal, 1:500, Cell Signaling cat# 8242S, molecular weight 65 kDa); Cox-2 (rabbit monoclonal, 1:500, Cell Signaling cat# 12282S, molecular weight 75 kDa); VE-cadherin (Cdh5; adherens junction (AJ) protein; mouse monoclonal, 1:100, Santa Cruz biotechnology cat# sc-52751, molecular weight 60 kDa); Claudin-5 (Cld5; tight junction (TJ) protein; mouse monoclonal, 1:500, Invitrogen cat# 35–2500, molecular weight 20 kDa). Blots were then washed three times in TBS Tween20 (0.1%) and then incubated for 1 h at room temperature with horseradish peroxide-conjugated goat antibody to mouse or rabbit in 2.5% milk TBS. Following subsequent washes, immunoreactivity was detected using SuperSignalWest Dura chemiluminescence detection reagent (Thermo Scientific, Waltham, MA, USA), and images were collected using a digital imaging system (Azure Imager c600, VWR, Radnor, PA, USA). For normalization purposes, membranes were incubated with 0.125% coomassie stain for 1–2 min and washed three times for 5–10 min in destain solution [119,120]. Densitometry was performed using ImageJ version 1.4 software (NIH, Bethesda, MD, USA). For total proteins, the signal value of the band of interest is expressed as a ratio of the corresponding coomassie signal. For phospho proteins, the signal value of the band of interest is expressed as a ratio of the corresponding total protein signal. This ratio of expression for each band is then expressed as a percent of the control included on the same blot.

### 4.12. Statistical Analyses

Parametric statistical analyses were used to analyze our datasets based on the assumption that our data fit a normal distribution and satisfy the sample size for adequate statistical power. Ethanol self-administration (ethanol consumed), BALs, and lever responses during extinction and reinstatement were evaluated using repeated measures of two-way ANOVA with session (or time) as a within-subject factor and treatment (vehicle/valcyte) or sex as between-subject factors. Significant interactions were investigated using Sidak’s post hoc tests. Differences in protein expression, immunoreactive cells, and plasma cytokine levels were compared between control, vehicle, and valcyte rats within each sex using one-way ANOVAs, followed by Sidak’s post hoc tests. Statistical significance was accepted at *p* < 0.05.

## Data Availability

Data will be provided by the corresponding author upon request.

## Supplementary Materials

The following supporting information can be downloaded at: https://www.mdpi.com/article/10.3390/ijms241512233/s1.
