# Supplementary material for: Progenitor Cells Play a Role in Reinstatement of Ethanol Seeking in Adult Male and Female Ethanol Dependent Rats
Source: Int J Mol Sci. 2023 Jul 31;24(15):12233. doi: 10.3390/ijms241512233 (PMC10419311; doi:10.3390/ijms241512233)
Supplement: Supplementary file 1 [file ijms-24-12233-s001.zip › ijms-2315837-supplementary.pdf]

Progenitor cells play a role in reinstatement of ethanol seeking in adult male and female ethanol dependent rats

Hannah A. Nonoguchi[1], Michael Jin[1], Rajitha Narreddy[1], Timothy Wee Shang Kouo[1], Mahasweta Nayak[1], Wulfran Trenet[1] and Chitra D. Mandyam[1,2]

Supplementary Results and Figure

Female and male rats have higher active lever responses for ethanol during CIE compared with pre-CIE levels, and in males, valcyte treatment increased active lever responding during relapse

Active and inactive lever responses for ethanol (10% v/v) consumption was determined in female and male rats prior to the onset of vapor exposure and during vapor exposure weeks (**Figure S1a-b**). Repeated measures two-way ANOVA for active lever presses for ethanol (10% v/v) did not show a sex x CIE interaction ($F (1, 50) = 0.7$; $p = 0.5$) and main effect of sex ($F (1, 50) = 1.03$; $p = 0.3$), however showed main effect of CIE ($F (1, 50) = 65.1$; $p < 0.001$). These data indicate that both sexes increased lever responses during CIE. In addition, we report rats that were given valcyte or vehicle did not differ in lever responses prior to the onset of vapor exposure and during vapor exposure weeks, indicating a non-biased separation of TK rats into each treatment condition ($p>0.05$; **Figure S1a**). Inactive lever responses did not differ between sexes and with CIE (ns; **Figure S1b**).

Two-way ANOVA of active lever presses during relapse revealed a sex x valcyte interaction ($F (1, 50) = 4.9$; $p = 0.03$), without main effect of sex or valcyte. Post hoc analysis revealed higher ethanol lever presses in vehicle females compared with vehicle males, and higher lever presses in valcyte males compared with vehicle males ($p < 0.05$; **Figure S1a**). Two-way ANOVA of inactive lever presses during relapse did not reveal a sex x valcyte interaction or main effect of sex or valcyte (ns; **Figure S1b**).

Vehicle and valcyte rats experienced extinction sessions over six days. Data from females and males were analyzed separately. In females and males repeated measures two-way ANOVA of inactive lever responses did not detect any differences (**Figure S1c-d**).

Vehicle and valcyte rats experienced reinstatement of ethanol seeking triggered by ethanol context and cues. Data from females and males were analyzed separately. Paired t test did not detect any significant increases in inactive lever presses compared with the last day of extinction in vehicle treated female and male rats (ns; **Figure S1e**). Paired t test did not detect any significant increases in inactive lever presses compared with the last day of extinction in valcyte treated female and male rats (ns; **Figure S1e**).

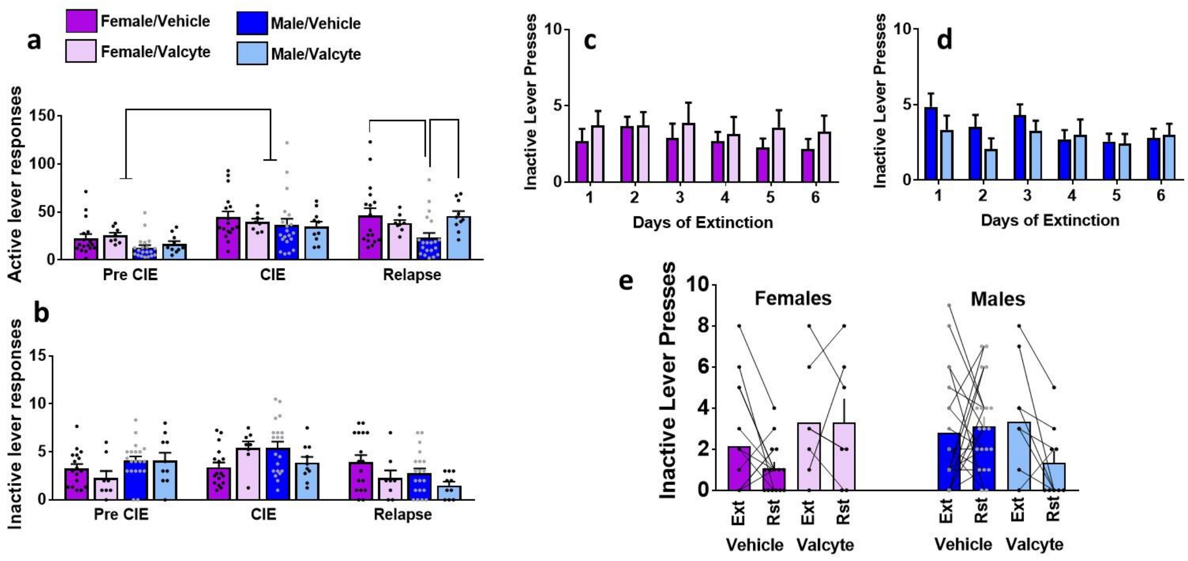

Supplementary Figure S1: Lever responses during pre-CIE, CIE, relapse, extinction and reinstatement sessions. Data indicate that active lever responses increased during CIE in males and females (a). Inactive lever responses did not differ between sexes and between sessions (b). Inactive lever responses did not differ between vehicle and valcyte groups during extinction sessions in females (c) and males (d). Inactive lever responses did not differ between extinction day 6 and reinstatement sessions in vehicle and valcyte groups in females and males (e). Significance is indicated by lines. Lines with heads indicate overall ANOVA. Lines without heads indicate differences with posthoc. Data is expressed as mean ± S.E.M. and significance is set at $p<0.05$.
